# Cognition across the Lifespan: Investigating Age, Sex, and Other Sociodemographic Influences

**DOI:** 10.3390/bs11040051

**Published:** 2021-04-13

**Authors:** Emily S. Nichols, Conor J. Wild, Adrian M. Owen, Andrea Soddu

**Affiliations:** 1 Faculty of Education, Western University, London, ON N6A 3K7, Canada; 2 Brain and Mind Institute, Western University, London, ON N6A 3K7, Canada; cwild@uwo.ca (C.J.W.); aowen6@uwo.ca (A.M.O.); asoddu@uwo.ca (A.S.); 3 Department of Physiology and Pharmacology, Western University, London, ON N6A 3K7, Canada; 4 Department of Psychology, Western University, London, ON N6A 3K7, Canada; 5 Department of Physics and Astronomy, Western University, London, ON N6A 3K7, Canada

**Keywords:** cognition, aging, sex, cognitive decline, statistical modeling

## Abstract

Maintaining cognitive health across the lifespan has been the focus of a multi-billion-dollar industry. In order to guide treatment and interventions, a clear understanding of the way that proficiency in different cognitive domains develops and declines in both sexes across the lifespan is necessary. Additionally, there are sex differences in a range of other factors, including psychiatric illnesses such as anxiety, depression, and substance use, that are also known to affect cognition, although the scale of this interaction is unknown. Our objective was to assess differences in cognitive function across the lifespan in men and women in a large, representative sample. Leveraging online cognitive testing, a sample of 9451 men and 9451 women ranging in age from 12 to 69 (M = 28.21) matched on socio-demographic factors were studied. Segmented regression was used to model three cognitive domains—working memory, verbal abilities, and reasoning. Sex differences in all three domains were minimal; however, after broadening the sample in terms of socio-demographic factors, sex differences appeared. These results suggest that cognition across the lifespan differs for men and women, but is greatly influenced by environmental factors. We discuss these findings within a framework that describes sex differences in cognition as likely guided by a complex interplay between biology and environment.

## 1. Introduction

In 2020, roughly 22% of the world’s population was over the age of 65, a total of approximately 1.7 billion people [1]. The consequences of our aging population are many, including an increasing focus on maintaining cognitive health. In order to be able to evaluate different tools and treatments for addressing cognitive aging, it is important that we first have a clear understanding of how cognition changes across the lifespan. Additionally, because of the often-cited cognitive differences between women and men [2,3,4,5], we must characterize cognition in each population; if sex differences in cognitive abilities do exist, then men and women may respond differently to cognitive aging interventions.

In healthy individuals, cognitive abilities develop rapidly throughout childhood [6,7,8,9]. By 18, executive function is thought to be mature [10], although research suggests that some processes continue to develop in early adulthood [11]. Young adulthood is where most researchers agree that cognitive abilities peak; however, there is large variability within this period across different cognitive functions [6,11]. Mid to late adulthood is then characterized by a slow decline in most cognitive abilities [8,12,13,14,15,16], and while it can be problematic, this decline is considered part of healthy aging.

Differences in cognitive abilities between men and women are less clear; although several sex disparities in cognitive abilities appear to exist, recent studies have found these differences to be mediated by underlying factors related to gender, such as socio-cultural factors, rather than being inherent to biological factors of sex. For example, Krinzinger and colleagues [17] found that number processing advantages in boys were mediated by attitudes toward mathematics, and similar results have been found in young adults [18] and in middle-school girls [19]. Differences in verbal processing have been less clear, with some suggesting that they are due to variability in instruction and strategy [20,21], and others suggesting a hormonal link [22,23]. Reports of sex differences in age-related cognitive decline are largely thought to be the result of cohort effects [24,25,26], although others have found sex-specific links to a brain-derived neurotrophic factor [27] and brain metabolic activity [28]. Realistically, the truth likely lies somewhere in between, with a multifaceted interaction of biology and environment [28,29].

Finally, there are a number of sociodemographic factors known to affect cognition. For example, it is generally agreed that higher socioeconomic status (SES) predicts better performance on cognitive tasks [30,31]. Additionally, anxiety, depression, and substance abuse also have known detrimental effects on cognition, with higher levels of all three being associated with poorer cognitive outcomes [32,33,34]. Such factors also interact with sex; women tend to experience higher levels of anxiety [35] and depression [36], while men experience higher levels of substance abuse [37], although women may be more at risk specifically for alcohol abuse [38] (but see [39]). Thus, there is a complex interaction of age, sex, and other sociodemographic variables that must be considered when studying cognitive abilities across the lifespan [40].

The internet provides a unique opportunity for examining cognition across the lifespan in the general population on a huge scale, allowing data to be sampled from participants from a broad range of SES, geographical, and educational backgrounds. Leveraging the power of the internet provides us with a cross-sectional snapshot of both demographics and cognition from a larger and more diverse sample than would be possible to collect in the laboratory. We invited participants to take part in an online study consisting of 12 tasks that compose the Cambridge Brain Sciences battery (www.cambridgebrainsciences.com, accessed on 27 October 2010). This executive battery assesses aspects of inhibition, executive function, selective attention, reasoning, verbal short-term memory, spatial working memory, planning, and cognitive flexibility, and three cognitive domain scores, namely working memory, verbal abilities, and reasoning, were calculated from the individual tests.

The present study had several goals. (1) The first goal of the present study was to characterize cognitive abilities across the lifespan, ranging from adolescence to late adulthood. Specifically, we sought to address whether differences exist between cognitive domains; do working memory, verbal abilities, and reasoning show the same pattern, or are they at their peak at different ages? Do they show the same rate of decline, or do some remain resilient to aging more so than others? (2) The second goal was to examine whether age effects differed between sexes, and what factors may influence these differences. Specifically, do sex differences exist in some cognitive domains and not others? Do men and women attain their highest scores at the same age, and do they decline at the same rate? (3) Further, we explored the demographic and social factors that affect the sexes differently, and whether controlling for these differences affects the observed pattern of cognitive abilities across the lifespan. Taking into account studies of the effects of mental health and sociodemographic variables on cognition, we predicted that: (1) the pattern of these abilities would show an increase up to early adulthood, and a slow decline into mid and late adulthood; (2) when not controlling for these factors, sex differences would manifest with men outperforming women in memory and reasoning, but with women outperforming men in verbal abilities; and (3) matching groups on these factors would eliminate sex differences in cognitive abilities.

## 2. Materials and Methods

### 2.1. Participants

All data for this study were collected with the Cambridge Brain Sciences (CBS) (www.cambridgebrainsciences.com, accessed on 27 October 2010) online platform, which has previously been used for other large-scale studies of cognition [41,42], and that has been validated for at-home use both in healthy controls and in older adults with Parkinson’s disease [43,44]. From a database of 76,452 participants, data from the participants who completed all questionnaire items and all 12 tests were included in analysis. A total of 65,994 participants (86.3%) met these requirements, with 13.7% (16.7% female and 12.5% male) withdrawing or providing incomplete data. Test scores were then filtered for outliers in two passes: scores greater than six standard deviations were assumed to be technical errors and were first removed. Then, scores greater than four standard deviations from the recalculated mean were identified, assumed to be performance outliers, and removed. Finally, individuals younger than 12 and older than 69 were removed because of low numbers outside of this age range, with 45,779 participants remaining. Two tightly matched samples of men and women were then created, with 9451 participants in each based on the number of participants that were able to be matched across groups. A summary of the sample’s demographics is included in Table 1. All participants gave informed consent, and ethics approval was obtained through the local Research Ethics Committee (2010.62).

A second set of analyses were run on the full dataset of 45,779 participants to determine what differences may exist in a sample that is reflective of the sociodemographic variance we see in the population. Descriptive information for these two new samples is summarized in Table S1. Scores are plotted against age in Figure S1, and histograms of domain scores are shown in Figure S2.

### 2.2. Materials

#### 2.2.1. Sociodemographic, Lifestyle, Psychological, and Sleep Questionnaire

The sociodemographic, lifestyle, psychological, and sleep questionnaire included questions about the individual’s age and sex, lifestyle such as exercise, substance use, and sleep, mental health such as depressive symptoms and anxiety, and other information such as education, employment, and level of technical savviness. When these data were collected, data were only collected on sex (male/female) and not gender; therefore, we do not have information on non-binary individuals. Data included in the present study are listed in Table 1. The questions used in the present study are included in the Supplementary Material.

#### 2.2.2. Cognitive Battery

Prior to filling in the questionnaire, participants completed the 12 tests in the CBS battery. The test order was fixed across participants. Detailed descriptions of the tests can be found in the Supplementary Material and in Table S2, but in brief they are: (1) ‘Monkey Ladder’ (visuospatial working memory); (2) ‘Grammatical Reasoning’ (verbal reasoning); (3) ‘Double Trouble’ (a modified Stroop task); (4) ‘Odd One Out’ (deductive reasoning); (5) ‘Spatial Span’ (working memory); (6) ‘Rotations’ (mental rotation); (7) ‘Feature Match’ (feature-based attention and concentration); (8) ‘Digit Span’ (verbal working memory); (9) ‘Spatial Planning’ (planning and executive function); (10) ‘Paired Associates’ (shape-location associative memory); (11) ‘Interlocking Polygons’ (visuospatial processing); and (12) ‘Token Search’ (working memory and strategy).

### 2.3. Procedure

Data were collected via the Cambridge Brain Sciences online platform (www.cambridgebrainsciences.com, accessed on 27 October 2010). The accuracy of online data has been found to be high [45], and this particular platform has been used in previous large-scale studies [41,42,46]. The experiment URL was originally advertised in a New Scientist feature, on the Discovery Channel web site, in the Daily Telegraph, and on social networking web sites including Facebook and Twitter. The 12 tasks were presented in a fixed order (note, the behavioral components were unrelated to the task order) and on completion of the trial participants filled out a demographic questionnaire. After reaching the website, participants were asked to give informed consent and to register with an e-mail address. They were then asked to complete 12 cognitive tests measuring a broad range of cognitive abilities, including inhibition, selective attention, reasoning, verbal short-term memory, spatial working memory, planning, and cognitive flexibility. They next completed a detailed questionnaire inquiring about demographic and lifestyle items (available in Appendix S1). This testing period took approximately 35 to 40 min.

### 2.4. Factor Analysis

The 12 tests were used to create three “composite” scores reflecting performance based on a previous factor analysis described in Hampshire et al. (2012). The three composite scores, labeled as working memory (WM), reasoning, and verbal abilities, were calculated as follows. First, the individual test scores were normalized (*M* = 0.0, *SD* = 1.0). Then, the three cognitive domain scores were calculated using the formula *Y* = *X*(*Ar*^+^)^T^, where *Y* is the N × 3 matrix of domain scores, *X* is the N × 12 matrix of test z-scores, and *Ar* is the 12 × 3 matrix of varimax-rotated principal component weights from Hampshire et al. All 12 tests contributed to each domain score, as determined by their component weights.

### 2.5. Statistical Analyses

Data were analyzed in R (version 3.5.2) [47] and RStudio (version 1.1.463). Specific packages included: ‘Segmented’ [48] for computing regressions with breakpoints, ‘MatchIt’ [49] for matching samples on demographic variables, ‘parallel’ for parallel computing, and ‘boot’ [50] for calculating confidence intervals. Figures were produced using ‘ggplot2’ [51]. Two groups of 9451 men and 9451 women were created, matched on with the nearest-neighbor matching method for all variables listed in Table 1. The nearest-neighbor matching method functions by matching without replacement based on a propensity score estimated using a logistic regression of the treatment on the covariates [49].

**Figure 1:**
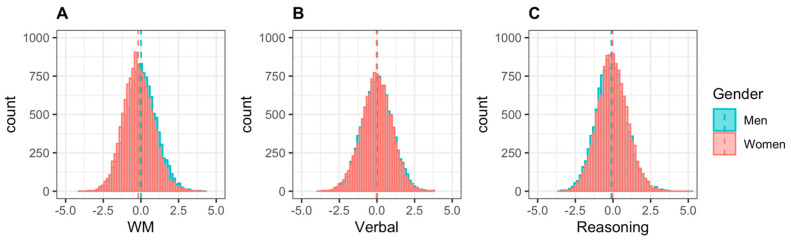
Histograms of domain scores by gender for (**A**) Working Memory, (**B**) Verbal, and (**C**) Reasoning. Dashed lines indicate mean.

To examine the differences in demographic variables between sexes, three different tests were used: Welch’s *t*-tests for continuous variables, Wilcoxon Rank Sum tests for ordinal variables, and chi-square tests for categorical variables. *p*-values were corrected for multiple comparisons using a false discovery rate and were considered significant at *p* < 0.01. Effect size was calculated using the appropriate measures for each test: Cohen’s *d* for *t*-tests, *r* for Wilcoxon Rank Sum tests, and Cramer’s *V* for chi-square tests. Measures of skew and kurtosis indicated that domain scores were normally distributed, and histograms are shown in Figure 1.

Segmented linear regression models were constructed to predict each of the three domain scores from participants’ reported age and were estimated using maximum likelihood estimation. Segmented regression was used to fit a model in which there is a change in the linear relationship—such as a “peak” that indicates a transition from increasing to decreasing performance across different ages—without imposing a pre-determined shape (e.g., quadratic or cubic) through adding one or more piecewise linear relationships [48,52]. The value of the independent variable (i.e., age) at which this change occurs is referred to as a breakpoint. The relationship between cognitive performance and age was modeled separately for each sex.

The segmented regression technique used here requires that the number of breakpoints, and (optionally) initial estimates of their locations, are provided. To determine the number of these points in each score, we fit each segmented regression model multiple times with one or more breakpoints and selected the model with the lowest Bayesian Information Criterion (BIC) [48,53]. The number of breakpoints was estimated separately for each domain score and sex. The algorithm converged on consistent breakpoint locations regardless of whether initial estimates were provided (from visual inspection of local regression curves, shown in Figure S3), or not. To confirm that a model with one or more breakpoints predicted the data better than a linear model, the Davies’ test [54] was used to determine whether there was a statistically significant change in slope. The estimated breakpoint location was taken as the age that was associated with peak performance in all regression models except for two cases. First, in men’s verbal scores, in which there were two breakpoints and the breakpoint with the highest score was used as the age at which performance peaked. Second, in women’s reasoning scores, in which the highest score was at the lower boundary of our age range. Slopes of the increasing and decreasing segments, as well as the middle segment for men’s verbal scores, were obtained using the ‘slope’ function of the ‘segmented’ package, and 95% confidence intervals (CIs) were calculated for peak age, score at peak age, and all slopes.

Differences in these parameters between men and women were analyzed by bootstrapping with 10,000 replications the difference of the estimated parameter values from models that were separately estimated for men and women. To determine whether these values differed significantly between sexes, the lower and upper 2.5% quantiles of the bootstrapped difference values were produced; if these bounds included zero, then it could be interpreted as no significant difference between the sexes.

**Figure 2:**
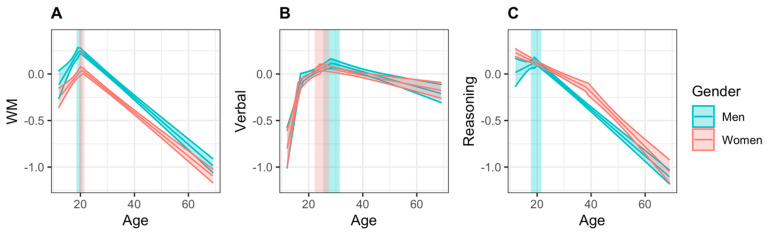
Regression lines for (**A**) Working Memory (WM), (**B**) Verbal, and (**C**) Reasoning scores across the lifespan, ranging from 12 to 69 years of age. The 95% simultaneous confidence bands are shown in translucent color around the line, and 95% confidence intervals for peak age are shown in translucent rectangles.

In segmented models where multiple breakpoints were deemed a better solution than a single point as determined using BIC, the increasing or decreasing portion of the curve (i.e., the data to the left or right of the “peak”) was characterized by two increasing or decreasing linear segments with different slopes (as can be seen in Figure 2C, women’s reasoning scores). In order to compare slopes between the sexes in these cases, bootstrapping was conducted by fitting the segmented model, then calculating the average slope to the left (in the case of men’s verbal scores) or right (in the case of women’s reasoning scores) of the peak. The rest of the bootstrapping parameters were kept the same as described above.

### 2.6. Secondary Analyses

Although matching groups on sociodemographic measures allows us to more accurately determine what the influence of sex alone is on cognitive performance, men and women do realistically differ on measures such as anxiety and sleep, and such factors are known to affect cognition. Thus, a second set of analyses were run on the full database (after cleaning of missing data and outliers, described below), to determine what differences may exist in a sample that is reflective of the sociodemographic variance we see in the population.

Local regression curves are shown in Figure S4. The same set of analyses were performed as outlined in the section above; however, because the total sample of men was larger than women, a random sample of 13,444 men were selected upon each bootstrap iteration in order to match the female sample size.

## 3. Results

### 3.1. Cognitive Domain Scores

#### 3.1.1. Working Memory

Results are reported in Table 2. A model with one breakpoint was found to best estimate women’s memory scores. The highest point in women’s WM scores occurred at age 20.42 (95% CI = 19.36, 21.48), with a score of 0.046 (95% CI = −0.009, 0.101). The slopes of the segments to the left and right of the breakpoint were 0.036 (95% CI = 0.019, 0.053) and −0.023 (95% CI = −0.025, −0.022), respectively, indicating that age was a significant predictor of WM performance in these age ranges; specifically, increasing age was associated with increasing scores up to the age of 20 years, after which it was associated with decreasing performance. Davies’ test for a change in slope was significant (*p* < 0.001), indicating that the linear relationship changed at the breakpoint, as can be seen in Figure 2A.

Men’s memory scores were also best estimated by a segmented model with one breakpoint. The highest point in men’s WM score occurred at age 19.65 (95% CI = 18.61, 21.48), with a score of 0.259 (95% CI = 0.187, 0.330). The slope of the increasing segment was 0.049 (95% CI = 0.022, 0.075), and the slope of the decreasing segment was −0.025 (95% CI = −0.027, −0.023), showing a significant effect of age on WM score in men. The change in slope was significant, as measured by the Davies’ test (*p* < 0.001). As can be seen in Table 3, there was no significant difference in the age at which women and men peaked in WM performance. However, men reached a significantly higher overall score than women at their peak ages, a difference of 0.21 standard deviations. When comparing how WM scores increased leading up to peak age and how quickly they declined afterward, women and men did not differ significantly.

#### 3.1.2. Verbal Abilities

Results of segmented regression of verbal scores are also summarized in Table 2. A model with two breakpoints was found to best estimate women’s verbal scores. Women first had a breakpoint at age 16.49, at which point the rate at which scores were increasing slowed (Figure 2B). The highest point in women’s verbal scores occurred at age 24.89 (95% CI = 22.26, 27.52) with a score of 0.071 (95% CI = 0.033, 0.108). The slope of the initial increasing segment was 0.153 (95% CI = 0.093, 0.214), the slope of the second increasing segment was 0.022 (95% CI = 0.009, 0.035), and the slope of the decreasing segment was −0.006 (95% CI = −0.008, −0.003), showing a significant relationship between age and verbal abilities. Davies’ test for a change in slope was significant (*p* < 0.001), indicating that the linear relationship changed at the breakpoint.

Men’s verbal scores were best estimated by a segmented model with two breakpoints. As can be seen in Figure 2B, men first had a breakpoint at age 17.16, at which point the rate at which scores were increasing slowed. The highest point in men’s verbal score occurred at age 28.42 (95% CI = 25.33, 31.52), with a score of 0.104 (95% CI = 0.050, 0.158). The slope of the initial increasing segment was 0.146 (95% CI = 0.094, 0.198), the slope of the second increasing segment was 0.015 (95% CI = 0.006, 0.023), and the slope of the decreasing segment was −0.008 (95% CI = −0.011, −0.005), indicating a significant relationship between age and verbal abilities in all three sections. The change in slope was significant, as measured by the Davies’ test (*p* < 0.001).

As summarized in Table 3, there were no significant differences in the age at which women and men’s scores reached a maximum in verbal abilities, scores at peak age, nor in the slopes of the increase and decrease in scores surrounding peak age.

#### 3.1.3. Reasoning

A model with one breakpoint was again found to best estimate women’s reasoning scores. However, this breakpoint occurred at age 38.12 years, and indicated a transition from a gradual to steeper decline: scores declined with a slope of −0.014 (95% CI = −0.017, −0.011) from age 12 to age 38.12, at which point the negative slope increased to −0.029 (95% CI = −0.035, −0.024). Davies’ test for a change in slope was significant (*p* < 0.001), indicating that the linear relationship changed. As can be seen in Figure 2C, the highest predicted scores for women occurred at age 12 with a score of 0.223 (95% CI = 0.187, 0.271). However, because this is the cut-off age of our sample, it is not possible to determine whether this is indeed a true peak, or if scores are higher at earlier ages.

Men’s reasoning scores were best estimated by a segmented model with one breakpoint. The breakpoint in men’s reasoning score occurred at age 19.62 (95% CI = 17.70, 21.54), with a score of 0.131 (95% CI = 0.060, 0.201). The change in slope was significant, as measured by the Davies’ test (*p* < 0.001); however, the slope of the initial segment was 0.015 (95% CI = −0.012, 0.041), and slope of the decreasing segment was −0.025 (95% CI = −0.027, −0.023), indicating that only the second segment showed a significant effect of age. Similar to women, this suggests that we did not capture a developmental increase in reasoning abilities within the current sample, and it is possible that the true peak occurs earlier than age 12.

Because we do not have a reliable measure of peak age in either sex, we compared between sexes the age at which reasoning scores began to decline. Women began to decline significantly earlier than men; however, reasoning scores at that age did not differ between sexes (Table 3). Because women did not show an increase in reasoning scores within our age range, we could not compare men and women on this measure. However, when comparing how scores declined after peak age, men declined significantly faster than women.

### 3.2. Unmatched Samples

Women and men differed on several demographic factors, but not for age, education, exercise, and number of siblings. While all significant *p*-values were ≤0.003, the largest effect sizes were seen in hours of sleep (Cohen’s *d* = 0.10), units of caffeine per day (Cohen’s *d* = −0.19), anxiety level (Wilcoxon’s *r* = 0.15), and technical savviness (Cramer’s *V* = 0.24).

#### 3.2.1. Working Memory

**Figure 3:**
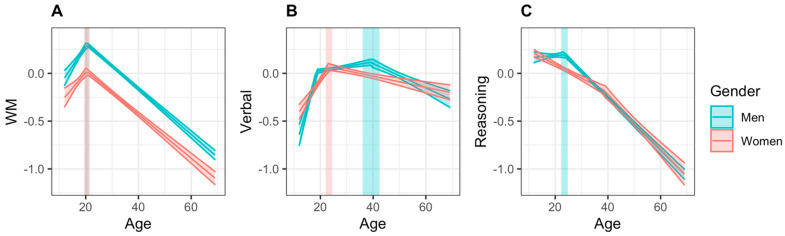
Regression lines for (**A**) Working Memory (WM), (**B**) Verbal, and (**C**) Reasoning scores across the lifespan, ranging from 12 to 69 years of age, in the socio-demographically unmatched sample. The 95% simultaneous confidence bands are shown in translucent color around the line, and 95% confidence intervals for peak age are shown in translucent rectangles.

Results of the segmented regression for WM scores of both sexes in the socio-demographically unmatched sample are reported in Table 4. Both women and men showed a significant change in slope as measured by the Davies’ test (*p* < 0.001 for both sexes). As can be seen in Table 5 and Figure 3A, no significant differences were found in the age at which women and men reached the highest point in WM, nor in the slopes of the increase and decrease in scores surrounding peak age. However, men reached a higher overall score than women at their peak ages by a standard deviation of 0.28.

#### 3.2.2. Verbal Abilities

Both women and men showed a significant change in slope as measured by the Davies’ test (*p* < 0.001 in all tests). A model with a single breakpoint best estimated women’s scores, while men’s scores were still estimated best by a model with two breakpoints. As summarized in Table 5, men reached the highest point in verbal abilities at a significantly later age than women. Men also had significantly higher scores at peak age, with a difference of 0.05 standard deviations. When comparing how scores increased up to peak age, women’s scores improved at a faster rate than men’s; however, there was no difference when comparing the rate of decline from peak age to age 69.

#### 3.2.3. Reasoning

Reasoning scores in our sample of women began to decrease at a significantly earlier age than men; however, scores at that age did not differ between sexes. While we did not capture an increase in reasoning abilities in either sex in our sample, reasoning scores decreased significantly faster in men than women (Table 5).

## 4. Discussion

After creating three cognitive domain scores from the 12 cognitive tests based on their underlying factor structure, we replicated previous findings that not all cognitive domains develop and decline in the same way [11,12]. Specifically, WM increased rapidly from age 12 to the early 20s, at which point it decreased at a steady rate until age 69, the upper limit of our sample’s age range. Verbal abilities also peaked in early adulthood, while reasoning did not show a clear peak in scores, instead being characterized by either a decline from age 12, or a plateau followed by a decline. These results are consistent with previous studies showing that cognition is not a unitary concept, and different cognitive abilities have separable developmental trajectories [11,12]. However, they extend the results of those studies in several important ways.

First, interpreting sex differences in cognitive data is complicated by the differences in socio-demographic factors. Several factors that were matched across groups, such as sleep and anxiety, have known effects on cognitive function [41], making it difficult to determine what is driving the observed sex differences in samples unmatched on these variables. Additionally, because these socio-demographic factors are sex-dependent, it is not possible to include them in the model due to issues with multicollinearity. By matching men and women on these factors, however, we were able to limit their effect on the data as much as possible, and this greatly reduced or eliminated the differences in cognitive performance and aging. Of course, there are numerous factors that we did not control for, such as reproductive health and occupation, and it is impossible to truly capture all of them. Finally, there are socio-demographic differences that may have biological underpinnings. For example, depression is more prevalent in women, perhaps due to the presence of sex-specific forms such as premenstrual dysphoric disorder [55]. It is therefore difficult to disentangle the environment from biological sex differences; however, accounting for these differences, regardless of their origin, is necessary for describing sex differences in cognition alone.

While these results are presumed to be reflective of the cognitive performance in a tightly controlled sample, when examining the progression of WM, verbal abilities, and reasoning in men and women in the broader database, all three cognitive domains showed unique differences. Although men and women’s scores reached peak WM performance at the same age, men reached a slightly higher score than women. In verbal abilities, women peaked faster and earlier, but men again reached higher scores. While women’s reasoning began to decline earlier than men’s, men declined at a faster rate. These results extend what is known from previous sex research. For example, there is evidence that men lose grey matter volume more rapidly with age than women, especially in fronto-temporal regions [56,57,58]; this in turn may lead to a faster decline in cognitive function, fitting the pattern observed here in the reasoning domain. In contrast, women are thought to have better verbal processing than men; however, we see the opposite here, with men reaching a higher peak score than women. One possible explanation for this discrepancy could be the age at which verbal abilities are tested. Burton and colleagues (19) tested a sample of university students, which is common in Psychology research. Looking at the pattern of verbal abilities in men and women in the current unmatched sample, women seem to outperform men at age 23, which, if we were to only examine individuals around this age, may lead to the erroneous conclusion that women have superior verbal abilities. Similarly, men are frequently reported to be better at mental rotation than women [22], a test included in our reasoning domain. Here, we found that peak reasoning scores did not differ between sexes, but women declined much earlier than men. Again, comparing sexes within a limited age range would have led to the erroneous conclusion that men outperform women in this domain, when in reality it is a difference in trajectory of reasoning abilities. The present results underline the need to take the progression of cognitive abilities across the lifespan into account when studying sex differences.

As noted above, creating broader groups in terms of sex-specific differences in socio-demographic factors increased the differences in cognitive performance and aging. In the case of WM, the sex difference between peak scores increased from 0.21 SDs to 0.28 SDs. Notably, differences in verbal abilities appeared, with women reaching a peak age significantly earlier, and men having a significantly higher peak score by 0.05 of one standard deviation. However, although the sex gap was smaller (or absent) in the matched sample, this does not mean that differences in the unmatched sample should be ignored. While they may not necessarily be inherent to biology, environmental influences are a part of life, and they do drive sex differences in cognitive abilities. Thus, it is reasonable to conclude that sex differences in cognition, based on biological sex alone, are minimal; however, there are notable effects of environmental factors that in turn drive sex differences in cognition. While exploring the relationships between individual sociodemographic factors and cognition was not the focus of the current study, future research should focus on elucidating these relationships.

One large area of disparity that remained even when controlling for environmental factors was with respect to the age at which reasoning abilities began to decline. Women declined significantly earlier than men, even when controlling for demographic factors. We were also not able to capture a reliable measure of the age at which reasoning abilities peak in either sex. In women, scores declined from 12 years of age. This could be because 12 is the age at which women’s reasoning abilities do indeed peak. However, it is also possible that women peak earlier, but due to a lack of data we were unable to determine the true peak from the current sample. Similarly, both unmatched and matched samples of men showed a plateau in reasoning scores until the point at which they began to decline. There are several possible explanations here. First, it is possible that men do peak in early adulthood, somewhere between 18 and 24 years of age, but the increase in reasoning abilities was not captured due to too small a sample size or noisy data. Second, they could follow a similar trajectory to women, with a slow decline before a steeper one, again not captured due to a lack of data. Because our sample of men was very large (over 32,000 in the unmatched sample), it is unlikely that either of these options are the case. Third, this plateau could be a true peak in reasoning, lasting several years, before beginning to decline. Previous research does suggest that reasoning abilities are relatively mature by age 12 [6,59], and another large-scale study has shown that by age 18, reasoning abilities have begun to decline [12]. Thus, although it is not possible to confirm that decline begins around age 12 in the current sample of women, the data follow a pattern that fits previous research and supports this claim.

The results presented here offer some insight into how to tailor interventions for cognitive decline appropriately for each sex. For example, women are known to experience more anxiety than men [35], a fact reflected in the current sample. Anxiety is known to correlate negatively with working memory [60]. Thus, to improve working memory, or protect against its decline, therapies should perhaps focus on reducing anxiety in everyone, with a targeted focus on women. Another example is substance abuse, which is more prevalent in men [37]. Because substance abuse negatively affects cognition [33], especially with respect to aging [61], a focused campaign aimed to reduce drug and alcohol consumption in men may yield a slowing in cognitive decline at the male population level. These sex-focused interventions can be combined with other treatments known to provide protection from cognitive decline, such as frequent exercise [62], for a well-rounded defence against cognitive aging.

There are a number of limitations of the present study that should be noted. First, the cross-sectional design does not allow us to control for cohort effects or historical bias. Second, there are a number of sociodemographic factors that were not included, and future research should aim to address their effects on aging and sex. Finally, because we did not collect data on gender, we cannot disentangle the relationship between biological sex and gender identity.

## 5. Conclusions

By examining a sample of over 45,000 individuals, ranging from 12 to 69 years of age, we showed how different cognitive abilities vary across the lifespan. Each domain had a unique relationship with age, demonstrating that not all cognitive processes follow the same pattern. Importantly, we found differences in the way women and men cognitively age, and showed that these disparities are reduced when controlling for socio-demographics such as sleep and anxiety. Nevertheless, some sex differences remained, supporting the notion that sex differences in cognition are likely guided by a complex interplay of both biology and environment.

## Figures and Tables

**Table 1:** Comparison of demographic variables across women and men in *N* = 18,902.

Measure	Mean (SD) or Percentage		χ^2^(df, N) or *t*(df)	*p*	Cohen’s *d*	BF_10_
	Women	Men				
*N*	9451	9451				
Age (years)	28.14 (10.95)	28.28 (10.65)	−1.31(23,696)	0.902	0.01	0.02
Highest education completed			10.18 (4, *N* = 18,902)	0.281	0.05	9.06 × 10^−5^
Some high school	9.70%	11.00%				
High School	8.30%	8.50%				
Some post-secondary	28.00%	27.50%				
Post-secondary degree	27.80%	27.10%				
Professional degree	26.10%	25.80%				
Level of employment			6.57 (5, *N* = 18,902)	0.902	0.04	4.76 × 10^−7^
No answer	3.70%	4.10%				
Unemployed	10.50%	11.40%				
Full time student	27.90%	27.60%				
Employed and student	14.90%	14.60%				
Employed part time	9.00%	9.20%				
Employed full time	34.00%	33.10%				
Exercise			4.07 (4, *N* = 18,902)	0.902	0.03	3.77 × 10^−6^
Never	10.40%	11.00%				
Infrequently	36.40%	36.90%				
Weekly	19.80%	19.80%				
Several times a week	26.60%	25.80%				
Every day	6.90%	6.50%				
Sleep (hours last night)	7.02 (1.62)	7.01 (1.63)	0.40 (18,899)	0.914	−0.01	0.02
Alcohol (units per week)	1.72 (1.76)	1.71 (1.76)	0.25 (18,900)	0.914	<−0.01	4.14 × 10^−23^
Caffeine (units per day)	3.47 (4.80)	3.52 (4.82)	−0.61 (18,900)	0.902	0.01	0.02
Cigarettes (per day)	1.53 (4.63)	1.68 (5.06)	−2.24 (18,749)	0.281	0.03	0.20
Depressive feelings			2.19 (5, *N* = 18,902)	0.914	0.02	1.35 × 10^−8^
No answer	1.10%	1.30%				
Never	10.90%	11.10%				
Occasionally	57.00%	56.60%				
Quite often	20.80%	20.60%				
Nearly every day	7.30%	7.40%				
All the time	3.00%	3.00%				
Anxiety			1.52 (5, *N* = 18,902)	0.914	0.02	1.50 × 10^−8^
No answer	1.20%	1.40%				
Never	14.00%	13.60%				
Occasionally	50.20%	50.30%				
Quite often	20.00%	20.20%				
Nearly every day	10.00%	9.90%				
All the time	4.50%	4.50%				
Tech savvy			0.02 (1, *N* = 18,902)	0.914	<0.01	0.02
Yes	76.80%	76.70%				
No	23.20%	23.30%				
Video games			4.67 (3, *N* = 18,902)	0.902	0.03	1.77 × 10^−4^
Never	33.80%	32.50%				
Monthly	26.50%	26.40%				
Weekly	23.50%	24.30%				
Daily	16.20%	16.80%				
Political leaning			1.29 (2, *N* = 18,902)	0.902	0.02	6.63 × 10^−4^
Liberal	47.40%	47.00%				
Middle	44.60%	44.60%				
Conservative	7.90%	8.40%				
Religiosity			0.97 (4, *N* = 18,902)	0.914	0.01	6.71 × 10^−7^
Atheist	33.50%	33.10%				
Agnostic	32.10%	32.10%				
Religious lapsed	18.70%	18.70%				
Religious practicing	11.90%	12.00%				
Very religious	3.90%	4.10%				
Siblings			2.30 (3, *N* = 18,902)	0.902	0.02	4.64 × 10^−5^
Only child	12.40%	12.40%				
Youngest	30.30%	30.50%				
Middle	16.50%	17.20%				
Oldest	40.80%	39.90%				

Note. Welch’s *t*-test used to compare numeric variables; all other tests used χ^2^.

**Table 2:** Segmented regression parameter estimates for age, from regression models estimated for each composite score.

Score	Gender	Term	Coef	SE	*t*	*p*
WM	Women	Age	0.04	0.01	4.10	<0.001
		∆Age	−0.06			
	Men	Age	0.05	0.01	3.61	<0.001
		∆Age	−0.07			
Verbal	Women	Age	0.15	0.01	7.58	<0.001
		∆Age1	−0.13			
		∆Age2	−0.03			
	Men	Age	0.15	0.03	5.36	<0.001
		∆Age1	−0.13			
		∆Age2	−0.02			
Reasoning	Women	Age	−0.01	0.001	−8.83	<0.001
		∆Age	−0.02			
	Men	Age	0.01	0.01	1.10	0.272
		∆Age	−0.04			

**Table 3:** Comparisons between genders matched on socio-demographic variables.

Score	Measure	Women (95% CI)		Men (95% CI)		Difference (95% CI)	
WM	Peak age	20.42	(19.36, 21.48)	19.65	(18.61, 20.69)	0.76	(−2.09, 4.32)
	Peak score	0.046	(−0.009, 0.101)	0.259	(0.187, 0.330)	−0.213	(−2.63, −0.159)
	Increase	0.036	(0.019, 0.053)	0.049	(0.022, 0.075)	−0.013	(−0.132, 0.028)
	Decrease	−0.023	(−0.025, −0.022)	−0.025	(−0.027, −0.023)	0.002	(−0.001, 0.005)
Verbal	Peak age	24.89	(22.26, 27.52)	28.42	(25.33, 31.52)	−3.53	(−20.49, 6.10)
	Peak score	0.071	(0.033, 0.108)	0.104	(0.050, 0.158)	−0.033	(−0.091, 0.019)
	Increase	0.035	(0.016, 0.048) ^a^	0.022	(0.006, 0.045) ^a^	0.013	(−0.012, 0.036)
	Decrease	−0.006	(−0.008, −0.003)	−0.008	(−0.011, −0.005)	0.002	(−0.003, 0.014)
Reasoning	Peak age	12		19.62	(17.70, 21.54)	−7.62	(−12.82, −2.23)
	Peak score	0.223	(0.187, 0.271)	0.131	(0.060, 0.201)	0.092	(−0.047, 0.151)
	Increase	–		0.015	(−0.012, 0.041)	–	
	Decrease	−0.020	(−0.021, −0.018) ^a^	−0.025	(−0.027, −0.023)	0.005	(0.003, 0.008)

^a^ Combined slope across two segments is reported. Note: Confidence intervals that do not include 0.0 represent a significant difference from 0.0 (*p* < 0.05 uncorrected).

**Table 4:** Segmented regression parameter estimates for age, from regression models estimated for each composite score, for models estimated with *N* = 45,779.

Score	Gender	Term	Coef	SE	*t*	*p*
WM	Women	Age	0.03	0.01	3.83	<0.001
		∆Age	−0.05			
	Men	Age	0.04	0.01	6.48	<0.001
		∆Age	−0.07			
Verbal	Women	Age	0.04	0.01	8.16	<0.001
		∆Age	−0.05			
	Men	Age	0.10	0.01	8.44	<0.001
		∆Age1	−0.09			
		∆Age2	−0.02			
Reasoning	Women	Age	−0.01	0.001	−9.62	<0.001
		∆Age	−0.01			
	Men	Age	0.003	0.004	0.73	0.468
		∆Age	−0.03			

Note: *p*-values for change in slope measured by Davies’ test; ∆Age refers to change in age parameter after the breakpoint.

**Table 5:** Comparisons between genders on key measures of cognitive performance over the lifetime, for models estimated with *N* = 45,779.

Score	Measure	Women (95% CI)		Men (95% CI)		Difference (95% CI)	
WM	Peak age	20.47	(19.39, 21.55)	20.48	(19.85, 21.12)	−0.01	(−4.70, 3.44)
	Peak score	0.021	(−0.007, 0.049)	0.304	(0.286, 0.323)	−0.283	(−0.331, −0.219)
	Increasing slope	0.032	(0.015, 0.048)	0.042	(0.029, 0.054)	0.010	(−0.071, 0.036)
	Decreasing slope	−0.023	(−0.025, −0.021)	−0.024	(−0.025, −0.023)	0.001	(−0.002, 0.005)
Verbal	Peak age	23.21	(22.00, 24.42)	39.20	(35.99, 42.42)	−15.99	(−26.36, −3.86)
	Peak score	0.067	(0.033, 0.101)	0.116	(0.074, 0.157)	−0.049	(−0.145, −0.002)
	Increasing slope	0.042	(0.032, 0.052)	0.014	(0.007, 0.027) ^a^	0.028	(0.012, 0.176)
	Decreasing slope	−0.006	(−0.008, −0.004)	−0.013	(−0.017, −0.009)	0.007	(−0.001, 0.019)
Reasoning	Peak age	12		23.51	(22.25, 24.78)	−11.51	(−16.96, −4.22)
	Peak score	0.208	(0.168, 0.249)	0.196	(0.163, 0.228)	0.012	(−0.136, 0.046)
	Increasing slope	–		0.003	(−0.004, 0.010)	–	
	Decreasing slope	−0.019	(−0.021, −0.018) ^a^	−0.027	(−0.029, −0.026)	0.008	(0.004, 0.012)

Note: Values are missing for women’s reasoning increasing slope as both segments were negative. Confidence intervals that do not include 0.0 represent a significant difference from 0.0 (*p* < 0.05 uncorrected). ^a^ Combined slope across two segments is reported. Slopes of the individual segments are reported in-text.

## Data Availability

The data that support the findings of this study are openly available in OSF at https://osf.io/wkyvn.

## Supplementary Materials

The following are available online at https://www.mdpi.com/article/10.3390/bs11040051/s1, Appendix S1: Sociodemographic, lifestyle, and sleep questionnaire, Appendix S2: Test Descriptions, Figure S1: All 45,779 raw data points across age for (A) Working Memory (WM), (B) Verbal, and (C) Reasoning cognitive domains, Figure S2: Histograms of cognitive domain scores by gender for (A) Working Memory (WM), (B) Verbal, and (C) Reasoning cognitive domains, in *N* = 45,779, Figure S3: Local regression curves for (A) Working Memory (WM), (B) Verbal, and (C) Reasoning scores across the lifespan, ranging from 12 to 69 years of age, in *N* = 18,902, Figure S4: Local regression curves for (A) Working Memory (WM), (B) Verbal, and (C) Reasoning scores across the lifespan, ranging from 12 to 69 years of age, in *N* = 45,779. Table S1: Comparison of demographic variables across women and men in *N* = 45,779, Table S2: Test—retest reliability (Pearson’s correlation, *r*) for all tests in the CBS battery calculated from a separate dataset provided by www.cambridgebrainsciences.com.
